# The Mediterranean Diet and Nutritional Adequacy: A Review

**DOI:** 10.3390/nu6010231

**Published:** 2014-01-03

**Authors:** Itandehui Castro-Quezada, Blanca Román-Viñas, Lluís Serra-Majem

**Affiliations:** 1 Department of Clinical Sciences, Health Sciences Faculty, University of Las Palmas de Gran Canaria, Luis Pasteur s/n, Las Palmas de Gran Canaria 35016, Spain; E-Mail: itandehuicastro@correo.ugr.es; 2 CIBER Fisiopatología Obesidad y Nutrición (CIBEROBN, CB06/03), Instituto de Salud Carlos III, Spanish Government, Madrid 28029, Spain; E-Mail: blancaRV@blanquerna.url.edu; 3 Community Nutrition Research Centre of the Nutrition Research Foundation, University of Barcelona Science Park, Barcelona 08028, Spain

**Keywords:** Mediterranean diet, dietary patterns, nutrient adequacy, requirements, micronutrient intake

## Abstract

The Mediterranean dietary pattern, through a healthy profile of fat intake, low proportion of carbohydrate, low glycemic index, high content of dietary fiber, antioxidant compounds, and anti-inflammatory effects, reduces the risk of certain pathologies, such as cancer or Cardiovascular Disease (CVD). Nutritional adequacy is the comparison between the nutrient requirement and the intake of a certain individual or population. In population groups, the prevalence of nutrient inadequacy can be assessed by the probability approach or using the Estimated Average Requirement (EAR) cut-point method. However, dietary patterns can also be used as they have moderate to good validity to assess adequate intakes of some nutrients. The objective of this study was to review the available evidence on the Nutritional Adequacy of the Mediterranean Diet. The inclusion of foods typical of the Mediterranean diet and greater adherence to this healthy pattern was related to a better nutrient profile, both in children and adults, with a lower prevalence of individuals showing inadequate intakes of micronutrients. Therefore, the Mediterranean diet could be used in public health nutrition policies in order to prevent micronutrient deficiencies in the most vulnerable population groups.

## 1. Introduction

The Mediterranean diet is known to be one of the healthiest dietary patterns [1]. The Mediterranean diet is a plant-based pattern, where vegetables, fruits, cereals (preferably as whole grain), legumes, and nuts should be consumed in high amount and frequency. The Mediterranean dietary pattern (MDP) also includes moderate consumption of fish and shellfish, white meat, eggs, and dairy products. On the contrary, consumption of red meat, processed meats, and foods rich in sugars and in fats should be small in both quantity and frequency. The principal source of dietary lipids of the MDP is olive oil and an adequate daily intake of water should be guaranteed, as well as moderate consumption of wine is recommended. Seasonality, biodiversity, the use of traditional and local food products are also important elements in this pattern. In addition, the Mediterranean diet has also qualitative cultural and lifestyle elements, such as conviviality, culinary activities, physical activity, and adequate rest [2]. It encloses a beneficial fatty acid profile with a high content of monounsaturated fatty acids (MUFA) and a higher MUFA/saturated fatty acids (SFA) ratio than non-Mediterranean diets [3,4]. High consumption of dietary fiber [5], low glycemic index and glycemic load [6], anti-inflammatory effects [7], and antioxidant compounds [8,9], may act together to produce favorable effects on health status.

The Mediterranean Diet is associated with a lower incidence of mortality from all-causes [10,11,12], and is also related to lower incidence of cardiovascular diseases [13], type 2 diabetes [14], certain types of cancer [15], and neurodegenerative diseases [10,11].

Finding a dietary pattern that fulfills the nutritional requirements of a population is a priority in order to establish nutritional recommendations [16]. Nutritional adequacy is defined as the sufficient intake of essential nutrients, needed to fulfill nutritional requirements for optimal health. According to the criterion of adequacy defined, the requirement for a given nutrient may be at a lower or higher intake amount. The criteria that are generally used to define adequacy of intake are: the prevention of deficiency diseases, the prevention of chronic diseases or the reduction of risk for diet associated diseases, subclinical nutritional health conditions identified by specific biochemical or functional measures, or requirements to maintain physiological balance [17]. Nutritional adequacy emerges from the comparison between the nutrient requirement and the intake of a certain individual or population. As neither the real intake nor the real requirement for one individual is known, the assessment of nutrient intake adequacy of an individual or population is based on the probability of adequacy [16].

The Mediterranean diet used to be sufficiently caloric and rich in vitamins and minerals, derived from vegetables and fruits, whole-meal cereals, nuts, virgin olive oil and fish, which made the risk of deficient micronutrient intakes quite infrequent. This explains why inadequate intakes of the B group vitamins (B1, B2, niacin, B6, folates, or B12) were rare in the Mediterranean basin, and intakes of antioxidant vitamins (vitamins E and C) and carotenes were also high [18,19]. However, people from Mediterranean countries are changing the traditional Mediterranean diet and include low nutrient dense foods (such as sugared soft drinks, sweets, bakery products, salted snacks) or vary their food processing methods (such as refinement of flour) towards a less healthy diet. These changes may have contributed to an increased risk of deficient intakes for some vitamins, especially folates, vitamins A and D, as well as inadequate intakes for the rest of the vitamins, in particular among certain population groups or collectives [18,19].

Nutritional adequacy may be used to determine the risk of deficiency of the nutrient assessed, in terms of low intakes or high intakes (for instance, the adverse effects of high levels of sodium intake may be applicable to reducing the risk of certain chronic diseases or conditions, such as hypertension) [20]. However, the complexity of the relationships between dietary intake and the pathology cannot be attributed to a single nutrient but, rather, to multiple nutrients and foods. Thus, the correct exposure has to be measured to understand such a relationship, and not only nutrients, but also foods, and the interaction between them, are of concern for this kind of evaluation. Food pattern analysis, such as the MDP, is then a key issue to investigate the linkages between nutrition and disease [21].

The objective of this study was to review the available evidence on the Nutritional Adequacy of the Mediterranean diet, its assessment, the general nutritional adequacy in different European and Mediterranean countries, and compared to a Western dietary pattern, as well as in children.

## 2. Methods

A scientific literature search was conducted on MEDLINE (National Library of Medicine, Bethesda, MD, USA) for relevant articles about the Mediterranean diet and nutritional adequacy published from January 2000 to June 2013. We used the keywords “Mediterranean diet”, “pattern”, “adequacy”, “nutritional”, “nutrient”, “intake”, “assessment”, and combinations, such as “Mediterranean diet and nutritional adequacy” or “Mediterranean diet and nutrient adequacy”. We narrowed the search to studies published in English, and limited to those conducted in humans. We focused the search on articles referring to the Mediterranean diet as a whole and excluded studies regarding specific foods of this diet. We limited the search to studies published in English and to those conducted in humans. Additional publications were identified from references provided in original papers. Only 15 articles were finally selected for this review.

### Nutritional Adequacy Assessment

The quality of the diet can be estimated in terms of food or food group intakes and diet patterns, or in terms of nutrient intake and the level of compliance with the nutrient requirements. When evaluating the diet in terms of nutrient adequacy, diverse types of analyses are used. The method used depends on the purpose of the analysis (to assess individuals or a population), on the nutrient under study and the type of distribution of the nutrient intake [22,23]. Recommendations used for the comparison will be country specific and evidence based.

**Figure 1:**
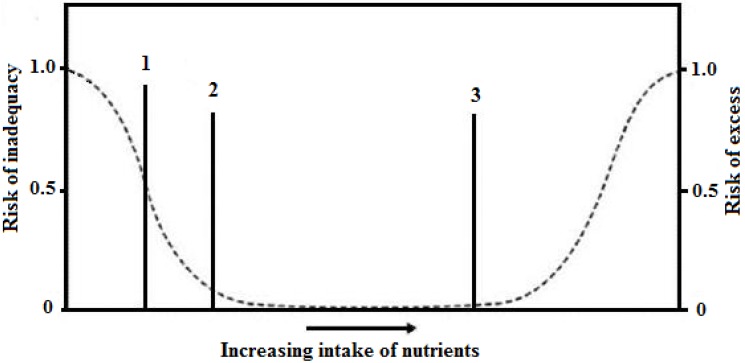
Graph of Nutrient intake values and the risk of nutrient inadequacy or excess ^a^. This image shows: (1) Average Nutrient Requirement (ANR); (2) Individual Nutrient Level (INL_x_); and (3) Upper Nutrient Level (UNL). ^a^ Adapted from*:* Institute of Medicine. *Dietary Reference Intakes: The Essential Guide to Nutrient Requirements*; National Academy Press: Washington, DC, USA, 2006 [20].

Most countries around the world recommend nutrient intake values for their populations but the amount of nutrients and the terms used to describe the requirement vary between them [24]. To avoid confusion, a standardized terminology of these recommendations has been proposed by a group of experts of the United Nations University (UNU), in collaboration with the Food and Agriculture Organization (FAO), the World Health Organization (WHO), and the United Nations Children’s Fund (UNICEF). The expert group proposed to use the Nutrient Intake Values (NIVs) as a common set of terms and definitions in all countries [24,25,26,27]. The NIVs encompass the following terms: the Average Nutrient Requirement (ANR), the Individual Nutrient Level (INL_x_), and the Upper Nutrient Level (UNL) (Figure 1) [28].

The Average Nutrient Requirement (ANR) is defined as the average or median usual intake value that is estimated to meet the requirement for a specific criterion in a life stage and gender group. The ANR is equivalent to the Estimated Average Requirement (EAR) used by the Institute of Medicine (IOM-USA). The INL_x_ is the recommended nutrient level for all healthy individuals in a specific subpopulation. The committees frequently add 2 SD to ANR, which will cover the needs of most of the population (*i.e.*, 98%), assuming that the distribution is symmetrical. The INL_98_ is equivalent to the Recommended Dietary Allowance (RDA) used by the IOM. Finally, most nutrients will have an Upper Nutrient Level (UNL), which is the highest intake that can be daily tolerated without risk of adverse health effects [28,29]. The term employed by the IOM is Tolerable Upper Intake Level (UL). Finally, an Adequate Intake (AI) is estimated if there is not enough scientific evidence to establish values for ANR and INL_x_. The AI has been included in IOM recommendations, but not in the standardized terminology proposed by the UNU [29]. These nutritional requirements are applied both to the nutritional assessment and to the planning of dietary interventions on an individual- and population-based level [16].

According to the IOM guidance, the prevalence of inadequate intakes for groups can be estimated by two methods: the probability approach and EAR (ANR) cut-point method. Regardless of the method actually chosen to estimate the prevalence of inadequacy, the ANR (or EAR) is the appropriate NIV to use when assessing the adequacy of group intakes [30,31].

The probability approach method requires the estimation of the probability of inadequate intakes for each individual in a population subgroup, averaging the probabilities, and then using this average as an estimate of the prevalence of inadequacy [23]. The EAR cut-point method measures the prevalence of inadequate intakes as the proportion of the population with usual intakes below the average nutrient requirement (ANR or EAR). It provides a good approximation of prevalence; however, it is necessary to fulfill the following conditions: intakes and requirements for the nutrient under study must be independent, the distribution of nutrient requirement must be distributed symmetrically, and the variance of the distribution of requirements should be smaller than the variance of the usual intake distribution [32].

Although certain nutrients are known to have a role in the etiology of several nutrient deficiencies and chronic diseases, the complexity of the relationship between dietary intake and disease cannot be reduced to the study of the effect that certain nutrients have on health. As such, not only nutrients, but also foods, and the interaction between them, are of concern for such an evaluation. Diet indexes (defined as a composite score of foods, nutrients, or both) were the first methods used in nutritional epidemiology to assess the effect that a combination of nutrients or foods may exert on health [22]. The Nutrient Adequacy Ratio (NAR) is an index of adequacy, which compares the individual’s daily intake of a nutrient with the INL_98_ for that nutrient [33]. Mean Adequacy Ratio (MAR) calculates the average for the Nutrient Adequacy Ratio values for the selected nutrients for a certain individual [33].

Diet indexes are known as *a priori*, as they are built based on dietary guidelines or recommendations. On the other hand, the *a posteriori* approach consists in defining food patterns once the dietary data are collected and using specific statistical analyses to identify the relevant actual food patterns of the study population [21]. Factor analysis or cluster analysis are the main statistical procedures used to analyze dietary data and identify dietary patterns [34]. Both *a priori* hypothesis-oriented diet indexes and *a posteriori* defined patterns have been related to the incidence of health outcomes (hard clinical end-points) and biomarkers in epidemiological or clinical studies. Some of these dietary patterns have been related to nutrient adequacy. This approach parallels a validation study, based on the rationale that if the classification of participants according to their adherence to the dietary pattern is able to determine whether or not they fail to reach the optimal nutrient intake, the use of the dietary pattern is sufficiently valid [21].

Some diet indices, *a priori* defined, have been correlated with the adequacy of certain nutrients for example, the revised Diet Quality Index (DQI) [35], Healthy Eating Index (HEI) [36], Dietary Diversity Score (DDS) [37], and the Food Variety Score (FVS) [38]. Similarly, the Mediterranean diet has been quantified in diet indices established a priori that attempt to make a global evaluation of the quality of the diet based on a traditional Mediterranean reference pattern [39]. For example, the Mediterranean Adequacy Index (MAI) was developed to assess how close a diet is to the Healthy Reference National Mediterranean Diet (HRNMD). Alberti *et al*. found that MAI values of diets in elderly participants from 10 European countries, followed for 10 years, were inversely associated with total mortality [40]. For children and youths, Serra Majem *et al*. developed the Mediterranean Diet Quality Index (KIDMED index) to assess the adequacy of the MDP in this age group [41,42].

Referring to the *a posteriori* defined analysis, the studies evaluating nutrient intake adequacy associated with dietary patterns showed that the Prudent pattern (defined by factor analysis as a diet rich in vegetables, fruits, legumes, whole grains, and fish) was valid to assess the intake adequacy of α-carotene, lycopene, and lutein, for men [43], and for assessing β-carotene, vitamin C, vitamin B6, and folic acid, for women [44].

Apart from the method used to identify dietary patterns, the micronutrients with less probability of being effectively assessed are vitamin B12 and vitamin E. Nevertheless, scientific evidence shows that some diet indices *a priori* or *a posteriori* defined are tools with fair to moderate validity to assess micronutrient intake adequacy [21].

## 3. Results

### 3.1. Prevalence of Nutritional Adequacy in Europe and Some Mediterranean Countries

Recently, Roman Viñas *et al*. estimated the prevalence of nutrient intake inadequacy in Europe using nutrient intake data already published [45]. The analysis of a number of micronutrients in adult and elderly European populations, showed a mean prevalence of inadequacy at or below 10% of the population for zinc, iron, and vitamin B12 (only in the elderly population); a prevalence between 11% and 20% for copper in the adult and elderly populations, for vitamin B12 in the adult population, and for vitamin C in elderly Europeans. Finally, micronutrients with a prevalence of inadequacy above 21% of the population were vitamin D, folic acid, calcium, selenium, and iodine, in the adults and elderly, and vitamin C in the adults only [45]. Previously Elmadfa *et al*. found no large differences of vitamin intake between four regions of Europe (North, South, Central-East, and West). However, the lowest intake levels of cobalamin and the highest intake levels of vitamin D were reported in the Northern region. Concerning the intake values of calcium, phosphorus, and iron, no large differences between the regions were observed. The intake of zinc was lower in the Western region than in the other regions and selenium intake was lower in the Northern region [46].

Another study conducted in eight European countries, evaluated the adequacy of nutrient intake in different age groups. Mensink *et al*. found that proportions below the EAR of calcium and copper were low. Inadequate intake of iodine was high in several countries, and for older adults in France and Germany the proportion below de EAR was 40%–60% [47]. Mean intakes of selenium were below the EAR in almost all countries, with high proportions with intakes below the EAR. Regarding iron, high proportions of inadequate intakes were found in teenage girls and women aged 11–50 years. Intakes of vitamin A, B1, B2, B6, B12, E, and C, were generally adequate. However, the proportions of the population with vitamin D intakes below the recommendations were exceptionally high, although the authors mention that in Mediterranean countries, vitamin D can be obtained from conversion through the skin stimulated by UV radiation, and therefore, the proportion that should be obtained from food is unknown. In Spain, more than 5% of intakes below the LRNI were found only for potassium, in women, and vitamin A, for elderly women [47].

The EURopean micronutrient RECommendations Aligned (EURRECA) Network of Excellence explored the process of setting micronutrient recommendations to address the variance in recommendations across Europe. Data on intake of vitamin C, vitamin D, vitamin B 12, folic acid, calcium, zinc, and iron (males only), from seven European countries were used for the assessment of inadequacy by applying the cut-point method. The highest ratios of inadequate intakes were found in countries such as Finland and Sweden, in males, and in Ireland and the United Kingdom, among females. In Mediterranean countries (Spain, Portugal, and Italy), the intake of calcium was in general adequate. In Spain, more than 20% of the male population presented three of seven vitamins and minerals with inadequate intakes, and for female population inadequate intake of four from six nutrients assessed was observed [17].

In the African Mediterranean countries the information is scarce. In Morocco, nutrient intake was evaluated among pregnant women. The mean daily intakes of energy and some nutrients were adequate. However, iron, folate, zinc, and calcium intakes were inadequate for the majority of women and more markedly in rural area [48]. On the other hand, Tunisian migrants living in the south of France, have also reflected better diet quality, variety, and adequacy than the local-born French, showing lower prevalence of chronic diseases compared with local-born French [49]. Information from other countries from the Maghreb and Middle East Mediterranean shows that, in Turkey, the average diet was inadequate to meet recommended daily intake of calcium, iron, riboflavin, vitamin A, and animal protein intakes in children. Deficient intakes of calcium, iron, and vitamin A were also found among adolescents and pregnant women [50]. In Iran, no national data on nutrient intake by age or sex groups are available, data is obtained through small surveys in many parts of the country. Results show that low-income families have low intakes of vitamins A and B_2_ (in some areas up to 50% and 70%, respectively). Iodine and iron deficiencies are the most frequent nutrient deficiencies in Iran [51,52].

As we have seen in this section, the nutrient intake varies according to the geographical zone. European countries have different nutrient intakes according to the culture, availability, and accessibility of food. It seems that among Europeans, countries in the Mediterranean basin have good nutrient intake quality, however, some countries of the Middle East Mediterranean and North Africa have severe deficiencies of essential nutrients. More research is needed on the adherence to the Mediterranean dietary pattern (MDP) and prevalence of nutrient adequacy in European and especially in Mediterranean countries.

### 3.2. Nutritional Adequacy of the Mediterranean and Western Dietary Patterns

The relationship between nutrient adequacy and the MDP and the Western dietary pattern (WDP) has been assessed in Spain by Serra-Majem *et al*. in a cohort study, Seguimiento de la Universidad de Navarra (SUN). The study assessed nutrient intake adequacy of certain vitamins (vitamins B12, B6, B3, B2, B1, A, C, D, and E) and minerals (Na, Zn, iodine, Se, folic acid, P, Mg, K, Fe, and Ca). The probability of intake adequacy for nutrients was estimated by the probability approach and using the NIVs [4].

The WDP was correlated with the intake of red and processed meat, eggs, sauces, precooked food, fast food, energy soft drinks, sweets, whole dairy, and potatoes, and showed a negative correlation with the consumption of low-fat dairy (Table 1). The food groups identified in the MDP were olive oil, poultry, fish, low-fat dairy, legumes, fruits, and vegetables (Table 1) [4].

**Figure 2:**
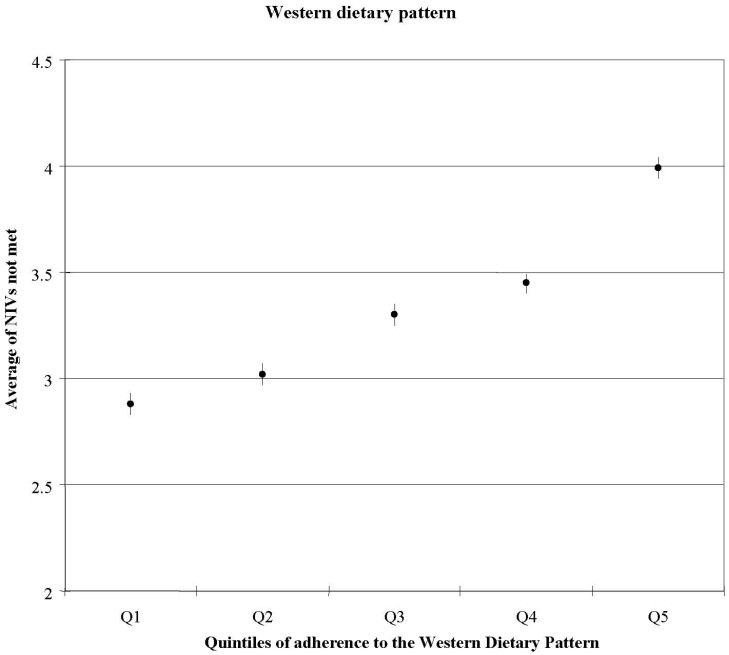
Average number of nutrients with intakes not meeting recommended levels across quintiles of Western Diet pattern score; adjusted for age and sex [4].

Subjects who scored high on the WDP were less likely to achieve adequate intakes of iodine, vitamin E, magnesium, iron, vitamin A, Se, vitamin C, and folic acid than those with a lower score. Furthermore, it was found that when the score on the WDP was high, the number of unmet nutrient intakes increased (Figure 2). Subjects in the highest quintile of WDP had a 2.5-fold increased risk for ≥10 NIVs unmet when compared to the lowest score on the WDP (OR: 2.48, 95% IC: 1.13–5.43, *p*-trend <0.001) [4].

**Table 1:** Correlation between baseline food consumption and factors representing the Mediterranean and Western dietary patterns in the Seguimiento de la Universidad de Navarra (SUN) cohort study (*n* = 17,197) [4].

Food groups *	Dietary Patterns **	
	Factor 1 (Western)	Factor 2 (Mediterranean)
Olive oil	-	0.32
Poultry	-	0.38
Red meat	0.54	-
Processed meat	0.5	-
Eggs	0.37	-
Fish	-	0.59
Sauces	0.42	-
Pre-cooked food	0.41	-
Fast food	0.57	-
Caloric soft drinks	0.35	-
Commercial sweets	0.4	-
Whole fat dairy	0.43	-
Low fat dairy	−0.31	0.37
Legumes	-	0.3
Vegetables	-	0.68
Fruits	-	0.54
Potatoes	0.45	-

* Presented in g/day; ** Correlation coefficients < 0.3 were omitted for simplicity.

**Figure 3:**
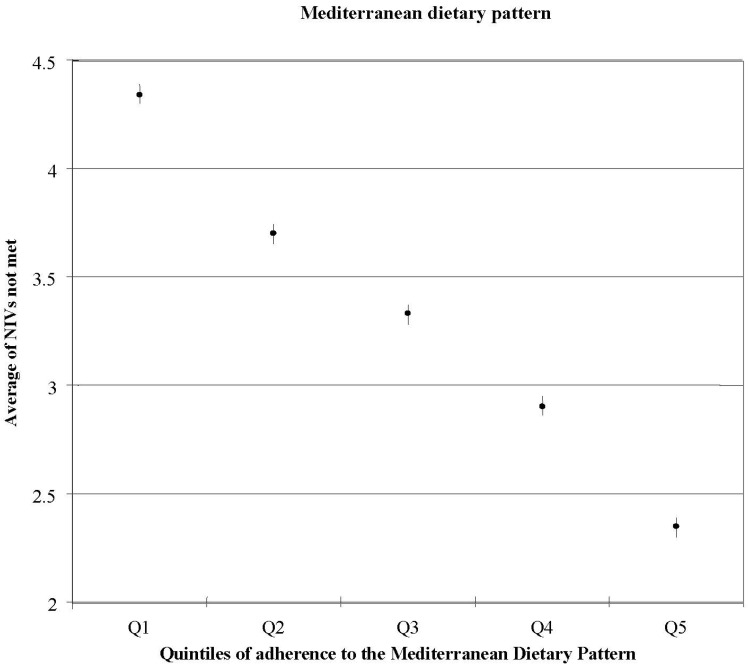
Average number of nutrients with intakes not meeting recommended levels across quintiles of the Mediterranean Diet pattern score; adjusted for age and sex [4].

Conversely, it was observed that higher scores of adherence to the MDP were associated with lower percentage of energy coming from total fat and SFA intakes. Ratio of MUFA to SFA increased with increased scores of adherence to the MDP (*p* for trend <0.001). Protein intake (as percentage of energy) increased across categories of adherence to the MDP [4]. Carbohydrate intake was low (43%–44%), showing a similar value across all the quintiles, on the contrary, consumption of dietary fiber increased according to the levels of adherence to the MDP. All the nutrients studied (except for sodium), showed increasing values with increasing scores to the MDP. Therefore, subjects with a higher score for the MDP had a better nutrient profile, with a lower prevalence of individuals showing inadequate intakes of micronutrients (Figure 3). People who scored high on the MDP were more likely to achieve adequate nutrient intakes of Zn, iodine, vitamin E, Mg, Fe, vitamin B1, vitamin A, Se, vitamin C, and folic acid, than those with a lower score. This population included premenopausal women and iron requirements are highly skewed due to menstruation, in order to address this issue, iron intake was log transformed for the estimation of nutritional adequacy. Therefore, according to their results, the MDP could address the potential risk of inadequate iron intake in women of reproductive age; however, more research is needed to confirm their results. Furthermore, it was found that subjects in the highest quintile of the MDP had lower risk for failing to meet ≥10 NIVs (OR: 0.02, 95% CI: 0.00–0.16, *p*-trend <0.001) when compared to the lowest category of adherence to the MDP [4].

Therefore, the MDP was directly associated with the MUFA/SFA ratio, showing a healthier profile of the quality of fat intake when comparing to other studies conducted in non-Mediterranean countries. Even more, as adherence to the Mediterranean diet increases, the probability of not fulfilling the nutrient recommendations decreases [4].

Another study conducted in 328 subjects (18–75 years) from Catalonia (Northeastern Spain), analyzed the association between different biomarkers and two Mediterranean diet (MD) adherence indexes. Bach-Faig *et al*. found that subjects with higher MD adherence had significantly higher plasma concentrations of beta-carotene, folates, vitamin C, alpha-tocopherol, and HDL cholesterol [53].

In France, Maillot *et al*. conducted a study by applying individual diet modeling in a representative sample of adults to evaluate the smallest dietary changes needed to fulfill a whole set of nutrient recommendations by each individual. Authors found that the inclusion of foods typical of the Mediterranean Diet were strictly necessary to achieve French nutrient recommendations [54].

### 3.3. Nutritional Adequacy in Children and the Mediterranean Diet

The Mediterranean diet has been associated with nutritional adequacy in Children. A cross-sectional study conducted in Spain, the enKid study, assessed individuals aged from six to 24 years (*n* = 3166) [55]. Information on dietary habits, lifestyle, and socioeconomic status was collected. To assess the compliance with a Mediterranean Diet model, a short questionnaire was used (KIDMED index), which allowed to classify subjects according to the quality of the Mediterranean Diet categorized as: High, Medium, or Poor (Table 2) [42]. The nutrient intake adequacy was assessed as the percentage of population with intakes below two-thirds of the recommended nutrient intakes.

**Table 2:** KIDMED test to assess the Mediterranean Diet adherence [41].

KIDMED test	Scoring
Takes a fruit or fruit juice every day	+1
Has a second fruit every day	+1
Has fresh or cooked vegetables regularly once a day	+1
Has fresh or cooked vegetables more than once a day	+1
Consumes fish regularly (at least 2–3/week)	+1
Goes >1/week to a fast food restaurant (hamburger)	−1
Likes pulses and eats them >1/week	+1
Consumes pasta or rice almost every day (5 or more per week)	+1
Has cereals or grains (bread, *etc*.) for breakfast	+1
Consumes nuts regularly (at least 2–3/week)	+1
Uses olive oil at home	+1
Skips breakfast	−1
Has a dairy product for breakfast (yoghurt, milk, *etc*.)	+1
Has commercially baked goods or pastries for breakfast	−1
Takes two yoghurts and/or some cheese (40 g) daily	+1
Takes sweets and candy several times every day	−1
**KIDMED Index**	**Adherence to the Mediterranean Diet**
Score ≤ 3 points	Poor
Score 4–7 points	Medium
Score ≥ 8 points	High

In the enKid study, the authors found that total energy intake did not change according to the KIDMED Index, with the exception of male adolescents aged 15 to 24 years, who showed a tendency towards increased levels. Consumption of fiber, calcium, iron, magnesium, potassium, phosphorus, and practically all the vitamins with the exception of vitamin E, increased according to the KIDMED Index. Results showed that the proportion of children with inadequate intake of calcium, iron (in females), magnesium, vitamin B6 (excluding males aged 6–14 years), vitamin C and A (in females), decreased when the scores of the KIDMED Index increased (Table 3) [41].

A study conducted in Spain showed that 20 healthy male adolescents aged 11–14 with a diet based on the MDP allowed to maintain adequate zinc serum levels despite the content of dietary phytate, which is present in vegetables, cereals and legumes [56]. In the same age group, there is evidence that when a MDP was consumed, a drastic increase in iron absorption was observed among the subjects when compared to their habitual diet [57]. Furthermore, in male adolescents another study has found significant increases in calcium absorption, calcium retention, and a considerable decrease in urinary calcium excretion with a Mediterranean type diet intervention when comparing to a basal diet [58].

**Table 3:** Percentage of inadequate intakes (<2/3 INL) in scholar children according to Mediterranean Diet adherence [41].

	KIDMED Index for 6–14 years			
	Poor ≤ 3 (%)	Medium 4–7 (%)	High ≥ 8 (%)	*p*-trend
Men/Women				
Energy	4.8/13.3	1.9/6.9	1.3/6.3	0.303/0.467
Protein	0.0/0.0	0.0/0.0	0.0/0.0	-
Calcium	4.8/26.7	2.6/10.4	0.4/4.2	0.027/<0.000
Iron	0.0/33.3	0.8/23.8	0.0/15.4	0.300/0.008
Magnesium	19.0/0.0	9.8/4.2	4.6/2.9	0.004/0.707
Thiamin	0.0/0.0	0.4/0.4	0.0/0.4	0.464/0.871
Riboflavin	0.0/0.0	1.1/1.2	0.4/0.8	0.559/0.881
Niacin	0.0/0.0	0.4/0.4	0.0/0.4	0.464/0.871
Vitamin B_6_	0.0/33.3	3.0/10.8	2.9/5.0	0.724/<0.000
Folate	14.3/46.7	9.8/32.3	5.0/23.8	0.021/0.010
Vitamin B_12_	0.0/0.0	0.0/0.0	0.0/0.0	-
Vitamin C	47.6/13.3	18/15.4	5.4/4.6	˂0.000/˂0.000
Vitamin A	57.1/80.0	63.9/61.5	59.6/54.2	0.523/0.024
Vitamin D	100.0/100.0	95.9/99.6	95.8/97.1	0.618/0.024
Vitamin E	28.6/66.7	43.2/60.8	36.3/57.1	0.394/0.310

## 4. Discussion

The Mediterranean diet has been associated with nutritional adequacy in adult population and children. Greater adherence to the MDP was related with a higher prevalence of individuals showing adequate intakes of micronutrients. The MDP had similarities with the healthiest patterns [43,44,59,60] defined in non-Mediterranean countries: a positive correlation with intakes of fruits, green leafy vegetables, poultry, and fish, and certain lifestyle habits, such as non-smoking and being more physically active [61]. However, when the association of the dietary patterns with their nutrient intake profiles was analyzed, differences arose, especially in relation to fat intake. Prudent and healthy patterns had lower intakes of total and saturated fat [44,62,63], and some studies found even lower intakes of MUFA [62,63]. Healthy patterns showed higher percentages of energy coming from proteins and carbohydrate and lower percentages of energy coming from fat [44,64] when compared the highest quintile to the lowest. Japanese traditional diets share a number of features with the Mediterranean diet in some food groups as cereals, beans, seafood, vegetables, and fruits. On the other side, both diets also differ in view of fat intake, where in Japan was extremely low in the past. Alcohol consumption is quite different due to the type of alcohol, one is wine and the other is sake or alcohol made of rice or corn [65]. Many of the characteristics of the diet in Okinawa are shared with the MDP, for example, the low intake of saturated fat, high antioxidant intake, and low glycemic load in these diets are likely contributing to a decreased risk for cardiovascular disease, some cancers, and other chronic diseases through multiple mechanisms, including reduced oxidative stress [66,67].

Therefore, the choice of the Mediterranean or the Japanese diet would be according to the circumstances and local availability, and trying to combine the best ingredients of both diets according to one’s habits and preferences. A new Japomediterranean diet that includes olive oil, wine, fish, beans, nuts and seeds, soya, vegetables, fruits, bread, rice, seaweed, dairy products, and mushrooms, could be the option for a promising future. Otherwise, keeping our own (Mediterranean or Japanese) traditional diet would be the best choice for our health and for our culture [67].

There are some limitations to this review, first, the evidence is very limited and almost all studies have been conducted in Spain. Second, to our knowledge, there are only few studies that relate nutrient intake of the MDP with biomarkers in adolescent population. Third, in the studies selected, the MDP was analyzed in different ways. Depending on how the Mediterranean diet is defined, the type of food and nutrient intake or other indices, such as glycemic index or glycemic load, could change. A more precise and quantitative definition of the Mediterranean diet is required if the adherence to this dietary pattern is intended to be accurately measured [39]. Therefore, more research is encouraged in order to give evidence-based recommendations on the Mediterranean diet and nutritional adequacy.

## 5. Conclusions

The Mediterranean Diet is a pattern with high nutritional quality; apart from better dietary fat quality [4], anti-inflammatory effects [7] and the increased quantity of antioxidants [68,69], we should also add the factor of enhanced nutritional adequacy. It has been demonstrated that higher levels of adherence to a Mediterranean dietary pattern are associated to a reduced risk of inadequate intakes. Therefore, health promotion strategies should be prioritized to promote the Mediterranean Diet, especially in population groups that are vulnerable to micronutrient deficiencies [32,70].
